# Evaluating the Presence of Marine Litter in Cetaceans Stranded in the Balearic Islands (Western Mediterranean Sea)

**DOI:** 10.3390/biology11101468

**Published:** 2022-10-06

**Authors:** Antònia Solomando, Francisca Pujol, Antoni Sureda, Samuel Pinya

**Affiliations:** 1 Research Group in Community Nutrition and Oxidative Stress, University of Balearic Islands, E-07122 Palma de Mallorca, Spain; 2 Interdisciplinary Ecology Group, Department of Biology, University of the Balearic Islands, E-07122 Palma de Mallorca, Spain; 3 Palma Aquarium Foundation, Carrer Manuela de los Herreros i Sorà 21, E-07610 Palma de Mallorca, Spain; 4 Health Research Institute of Balearic Islands (IdISBa), E-07120 Palma de Mallorca, Spain; 5 CIBER Fisiopatología de la Obesidad y Nutrición (CIBEROBN), Instituto de Salud Carlos III (ISCIII), E-28029 Madrid, Spain

**Keywords:** litter impacts, plastic ingestion, entanglement, cetaceans, western Mediterranean

## Abstract

**Simple Summary:**

The presence of plastic in oceans is extremely concerning as it poses a potential threat to marine organisms; for instance, they could become entangled in the plastic or they could ingest it. The objective of this work is to provide evidence, for the first time, of the impact that plastic debris has on stranded cetaceans in the Balearic Islands, in terms of ingestion and entanglement. We examined the occurrence of marine debris in the gastrointestinal tracts of 30 cetaceans, from five different species, that were found stranded around the Balearic Sea: *Stenella coeruleoalba*, *Tursiops truncatus*, *Grampus griseus*, *Balaenoptera physalus*, and *Physeter macrocephalus*. Three specimens (10% of the sample) were found to have ingested plastic items, including fishing nets, plastic bags, and strapping lines. The species affected were *T. truncatus*, and *P. macrocephalus*. Moreover, a total of seven cases of entanglement were recorded during the study, affecting four different species (*S. coeruleoalba*, *T. truncatus*, *P. macrocephalus*, and *Megaptera novaeangliae*), and all of them were entangled in discarded fishing nets. When possible, plastics were characterised by size, shape, colour, and polymer type. We concluded that the occurrence of marine debris observed in this work confirms the impact of plastic pollution on cetaceans in the Balearic Sea for the first time.

**Abstract:**

The global distribution and presence of plastic, at all levels of the water column, has made plastic debris one of today’s greatest environmental challenges. The ingestion and entanglement of plastic-containing marine debris has been documented in more than 60% of all cetacean species. In light of the increasing pressure on cetaceans, and the diversity of factors that they face, the aim of this work is to provide evidence of the impact of plastic debris on stranded cetaceans, in terms of ingestion and entanglement, in the Balearic Islands for the first-time. Detailed examinations, necropsies, and plastic debris analysis were performed on 30 of the 108 cetaceans stranded between 2019 and 2022. Specimens belonging to five different species, *Stenella coeruleoalba*, *Tursiops truncatus*, *Grampus griseus*, *Balaenoptera physalus*, and *Physeter macrocephalus*, were evaluated. Ten percent of the cetaceans (N = 3) presented plastic debris in their stomach, with one case of obstruction and perforation. Fishery gear fragments (ropes and nets) were found in two adults of *T. truncatus*, whereas packaging debris (plastic bag, packing straps, and plastic sheets) were found in a juvenile *P. macrocephalus*. Plastic items analysed by Fourier transform infrared spectroscopy (FT-IR) reported three polymer types: polypropylene, polyamide, and high-density polypropylene. A total of seven cases of entanglement were recorded during the study, affecting four different species (*S. coeruleoalba*, *T. truncatus*, *P. macrocephalus*, and *Megaptera novaeangliae*). Only two individuals were freed from the nets, although one died after a week, whereas the rest were already found dead. In conclusion, data collected in the present study provided evidence of plastic ingestion and entanglement in cetaceans of the Balearic Islands for the first-time, thus highlighting the need for the regular examination of stranded cetaceans (as they are top predators) in future research to better understand the effects of these pollutants.

## 1. Introduction

Marine litter, overrepresented by plastic elements, has emerged as a serious threat for major marine taxa, with well-documented negative effects on more than 1400 species [1]. For marine species, the impacts of plastic pollution can be divided into those arising from entanglement, which can result in direct injury, drowning, or strangulation, and those from ingestion, with pathologies ranging from malnutrition to gastrointestinal blockages [2,3,4,5]. To date, plastic ingestion has been recorded in 81 marine mammal species [6,7,8,9], all seven sea turtle species [10,11,12], and 203 seabird species to date [13], and these numbers are still increasing [14]. In marine mammals, the ingestion of litter can occur either directly, when an animal mistakes an item for prey, or indirectly, through the consumption of prey that contains waste [15,16]. The effects of ingestion are diverse, depending on species, the type of material, the amount ingested, and location. Debris in the forestomach can cause distention, obstruction, ulceration, perforation, and peritonitis, or they can functionally alter digestion, induce satiation, cause starvation, and general debilitation [8,15,17,18,19]. Moreover, ingested litter can contribute to chemically induced harm through the bioaccumulation of pollutants contained or absorbed by the litter, such as plasticizers, chemical additives [20], absorbed persistent organic pollutants (POPs) [21], and heavy metals [22], many of which are known neurotoxins or endocrine disruptors [23]. In cetaceans, the ingestion of litter has been documented in over 58% of all cetacean species, including species with different feeding strategies throughout the water column [24], and these numbers are increasing over time [14]. Furthermore, it has been reported that deaths attributed to litter ingestion can lead to mortality rates of up to 22% in stranded animals [25], suggesting that litter could be a significant conservation threat to cetaceans. Despite that fact, entanglement events have only been documented in ~30% of cetacean species [6], although there is evidence to suggest that population declines have been due to entanglement [26,27], even pushing some species such as the Vaquita (*Phocoena sinus*) towards extinction [28]. In 1987, Laist [29] reported that fishing gear (monofilament line, nets, and ropes) was the primary cause of entanglements. Most of this material originated from commercial fishing operations, although it was found that cargo ship equipment and recreational fishing equipment may also contain items that cause entanglement; however, it is usually difficult to distinguish what may cause entanglement in active fishing gear (by-catch) from what may cause entanglement in lost or discarded gear [25]. Generally, cetaceans tend be entangled around their neck, flippers, and flukes [30]. The impact of entanglement can be severe, ranging from death, death via starvation due to impaired swimming, laceration of large blood vessels as a result of foraging, amputations, and systemic infections that reduce animal fitness [31,32,33].

However, the number of records does not reflect the magnitude of the problem in cetaceans, due to low detection rate and difficulty in retrieving and analysing specimens [9,34]. This fact highlights that the cetaceans which are stranded along the coast provide a valuable opportunity to study the interactions between animals and marine litter. In this sense, rescue centres and stranding networks have become very important entities for collecting data and monitoring the external and internal wellbeing of stranded cetaceans.

Cetaceans are widely regarded as reliable sentinels of ocean health due to their position as top predator in the marine food web, their conspicuous nature, and their reliance on marine resources. Remarkably, no studies have been published on cetaceans and the impact of plastic pollution in the Balearic Islands (Western Mediterranean). This lack of data underlines the need to further engage with research efforts that aim to understand the magnitude of the issue of plastic pollution on cetaceans in the water surrounding the Balearic Islands; therefore, the current study focuses on ingestion and entanglement data, but the collected plastic items are nevertheless characterised to allow comparisons with earlier works. The main objective of the present work is to increase knowledge on the tendency of cetaceans to ingest plastic litter and/or become entangled in it in the waters of the Balearic Islands by monitoring stranded cetaceans. The second objective focuses on characterizing the variety of plastics ingested, and the variety of plastics that the cetaceans become entangled in, noting the size, shape, and colour of the plastics. The third objective aims to identify the most frequent polymer types that cetaceans ingest and become entangled in using FT-IR analysis. We hypothesize that plastics are likely to be a minor, albeit significant, cause of cetacean mortality in this area, either due to ingestion or entanglement, and that the less serious effects of plastic ingestion and entanglement remain poorly understood.

## 2. Materials and Methods

### 2.1. Area of Study and Species

**Figure 1:**
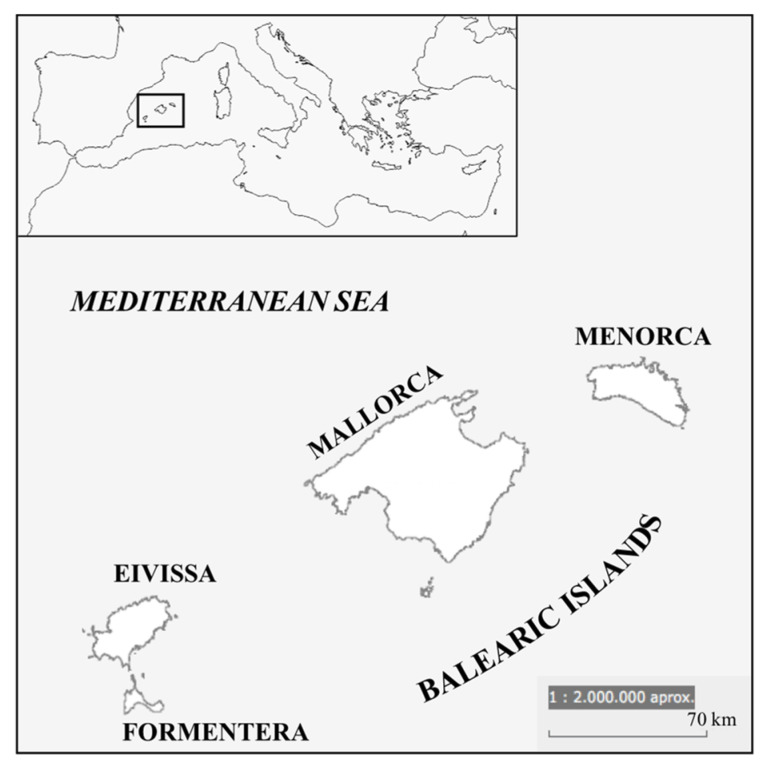
Map of the study area in the Balearic Islands. Names of the four principal islands are written in bold. Inset box indicates the location of the Balearic Islands in the western Mediterranean Sea.

The specimens of the cetaceans analysed in the present study were sampled between January 2019 and August 2022 along the coast of the Balearic Islands, which extends for more than 1700 km (Figure 1). The Balearic Islands are situated in the western part of the Mediterranean Sea and are formed of four inhabited islands (Mallorca, Menorca, Eivissa, and Formentera). The Balearic region has a residential population of 1,173,008 [35]; however, because these islands are a major tourism hub during the summer months, the seasonal peak, in terms of population, reaches more than two million people [35]. The seasonal presence of some cetacean species in the Balearic waters varies depending on migratory routes, but according to the scientific literature, we found 8 resident species [36], including 1 baleen whale (Mysticetes) (*Balaenoptera physalus*, the second-longest species of cetacean on Earth) and 7 toothed whales (Odontocetes) (*Physeter macrocephalus, Ziphius cavirostris, Globicephala melaena, Grampus griseus, Tursiops truncatus, Stenella coeruleoalba,* and *Delphinus delphis*). Notably, the killer whale (*Orcinus orca*) (Casinos, 1981), the false killer whale (*Pseudorca crassidens*) (Castell and Gutiérrez, 1991), and the humpback whale (*Megaptera novaengliae*) (Aguilar, 1989) have been registered. Three of the toothed whale species, *Physeter macrocephalus, Globicephala melaena*, and *Delphinus delphis* are currently listed as Endangered by the IUCN Red List on the Mediterranean IUCN Red List of Threatened Species [37], whereas *Balaenoptera physalus, Tursiops truncatus*, and *Stenella coeruleoalba* are listed as Vulnerable by the IUCN Red List in the Mediterranean [37]. Two species, *Grampus griseus* and *Ziphius cavirostris*, are considered to be data deficient in the Mediterranean, but are globally listed as being of Least Concern according to IUCN criteria [37].

### 2.2. Sample and Data Collection

All specimens were obtained using the sea turtle/cetacean stranding network of the Balearic Islands (Fundación Palma Aquarium Rescue Centre) during 2019–2022. Cetaceans were found stranded along the coastline or picked floating while adrift. Cetaceans found alive were immediately attended to by the veterinarian team, although unfortunately, in most cases, animals were finally euthanised due to poor prognosis. Carcasses from dead specimens were transported to the necropsy camera of the rescue centre for necropsy.

For each cetacean, clinical history, date and location of stranding, date of necropsy, species, gender, biometric data, and state of decomposition were systematically recorded in an annual database. Moreover, animals were classified into age classes according to [38], as follows: neonate (i.e., animals with vibrissae hairs, unhealed navel, foetal folds over the body, and soft and folded dorsal fin and tail flukes), calf (i.e., animal with presence of milk in its stomach, or about the size of a nursing calf), juvenile, (i.e., body length smaller than the adult but larger than a calf), and adult, (i.e., body length of an adult).

Post-mortem examinations of all cetaceans were carried out following the protocols of The Interreg Med Plastic Busters MPAs project [39]. When possible, specimens were measured (Total length (TL)) and sexed by a visual inspection of gonadal gross morphology, and they were examined for external lesions and other anomalies. Particular attention was paid to any signs that were indicative of direct interactions with fishing gear.

#### 2.2.1. Ingestion

The digestive tract (oesophagus, stomach chambers, and intestines) of stranded dead cetaceans were carefully dissected and analysed for the presence of plastic litter (Table 1). Only in one case (stranded *B. physalus*) was the necropsy was done in situ, on the beach in Mallorca, due to the animal’s large size and accessibility. In order to prevent external contamination during sampling, metallic clamps were fixed on the oesophagus, stomach, and intestine before removing their content. Moreover, dead specimens with perforations on the digestive tract were excluded from the analysis. Digestive parts were collected and stored at −20 °C until further analysis. Frozen organs were transferred to the Interdisciplinary Ecology Group laboratory at the University of the Balearic Islands for litter analysis. One day before content analysis in the laboratory, the digestive organs were left to thaw. Content examination was carried out following the protocols from the Plastic Busters MPAs project. Each part of the cetacean was weighed before and after opening, and the resultant content was carefully filtered through a set of two metallic mesh sieves (5 mm and 1 mm) to avoid mixing the content from the isolated sections. The ingested plastics were carefully washed with distilled water and allowed to dry at 40 °C in the 12 h before being properly examined and classified. In accordance with the protocol, each piece was counted and photographed, weighed (dry mass), and measured (maximum length). Furthermore, for each item, the colour was recorded, and the shape was categorised into one of four types [40]: sheet-like (e.g., plastic bags), thread-like (e.g., nylon lines, ropes), foams (e.g., polystyrene foam), and fragments (e.g., bottle cap). The colour categories were as follows: white–transparent, dark coloured (black, blue, dark green), and light coloured (cream, yellow, pink, light green). Plastic size was grouped in accordance with the criteria set out by Barnes et al. (2009) [41], which are as follows: macroplastics (>20 mm), mesoplastics (>5–20 mm), and microplastics (<5 mm). Furthermore, Fourier transform infrared spectroscopy (FT-IR) (Bruker OPTICS, Germany) was used to identify the synthetic polymers of the plastic items collected. Before FT-IR analysis, plastics were carefully rinsed with deionised water and dried, with the purpose of achieving precise spectra [42]. FT-IR measurements were carried out using an attenuated total reflectance (ATR) mode that had a wavelength range of 4000–400 cm^−1^, 16 co-added scans, and a spectral resolution of 4 cm^−1^. The ATR diamond and its base were cleaned with ethanol before and after the procedure and between every sample. All spectra were then compared with commercial and self-generated polymer libraries. Similarities greater than 70% of the Hit Quality Index were considered valid [43]. We analysed all the spectra using the spectroscopy software OPUS. The analysis was carried out with the support of the Scientific/Technical Services at the University of the Balearic Islands. Organic content such as wood fragments, pebbles, and food remains were collected but were excluded from the analysis.

All equipment used for the dissection and litter analysis was carefully cleaned with distilled water in order to avoid any possible contamination.

#### 2.2.2. Entanglement

Entanglement may be confused with by-catch, which is the ensnaring of non-target animals in active fishing apparatus. It may be difficult to diagnose whether the retrieved bodies were caught in active gear or lost gear, and marks on bodies may be misidentified as net marks when they were actually the result of entanglement in marine debris [9]. Given these difficulties, in this study, cases of entanglement have been considered in two ways: specimens in which gear was identified as likely having been operational at the time of entanglement; and specimens entangled in ghost fishing nets. In all cases, the type of material in which specimens have been entangled was classified according to the following categories: pieces of net, monofilament line (nylon), rope or pile of ropes, plastic bags, raffia, other plastics, multiple materials, or unknown. In addition, plastic pieces obtained from the specimens were characterised by FT-IR and were photographed.

Cetaceans with an advanced state of decomposition were not included in the entanglement analyses due to the impossibility of observing entanglement impacts, such as net marks on the animal’s body.

### 2.3. Statistical Analysis

The frequency of plastic occurrence (FO) was estimated as the percentage of the individuals examined that contained plastics in their digestive tract. Digestive content and entanglement information, together with biometric data, were registered in a Microsoft Excel (version 16.16.27) spreadsheet.

## 3. Results

Between 2019 and 2022, a total of 108 cetaceans, from nine different species, stranded along the coast of Balearic Islands were registered. Twenty individuals (18.5%) could not be identified at the species level due to the poor condition of the corpse. Of the remaining 88 specimens, the species recorded in order of abundance were: *S. coeruleoalba* (N = 44), *T. truncatus* (N = 26), *G. griseus* (N = 5), *P. macrocephalus* (N = 5), *G. melaena* (N = 3), *Z. cavirostris* (N = 2), *B. physalus* (N = 1), *Eschrichtius robustus* (N = 1), and *M. novaeangliae* (N = 1). The cause of stranding/death could only be determined in five cases, thus showing clear signs of interactions with fisheries (4.6%, 2 *S. coeruleoalba*, 2 *T. truncatus*, and 1 *P. macrocephalus*).

### 3.1. Ingestion Analysis

Detailed examinations and necropsies were performed on 30 (27.8%) of the 108 cetaceans, including 16 females, 11 males, and three individuals that were not sexed. These cetaceans belonged to five species: *S. coeruleoalba* (N = 15), *T. truncatus* (N = 9), *G. griseus* (N = 2), *B. physalus* (N = 1), and *P. macrocephalus* (N = 1). In two cases, the species could not be determined. For the rest of the specimens, it was impossible to study the digestive tract given the difficulty of transporting from their location, or the advanced state of decomposition in which some of these specimens were found. The summary of stranded and necropsied cetaceans by species, date, biometric data, age, gender, and digestive content information is shown in Table 1. Of these thirty individuals, five (16.7%) were euthanised due to poor prognosis, four (13.3%) were found alive but died shortly after stranding, six (20%) were found fresh or very fresh, and fifteen (50%) were found in a moderate or advanced state of decomposition.

The mean total length (±SD) of *S. coeruleoalba* was 1.9 ± 0.1 m. According to their body length, eleven were adults, three were juveniles, and one was a calf. Of these cetaceans, nine specimens were female, four were male, and three individuals could not be sexed due to the advanced state of decomposition. In *T. truncatus*, there were six adult specimens, two juveniles, and one calf, and the mean total length (±SD) was 2.4 ± 0.3 m. Furthermore, five specimens were male, three were female, and one individual could not be sexed. The mean total length (±SD) in *G. griseus* was 2.8 ± 0.4 m, the calculation for which was based on two females, one adult, and one juvenile. The *B. physalus* examined was a female adult of 15.5 m length. Finally, the *P. macrocephalus* analysed was a male juvenile with a total length of 5.4 m.

**Figure 2:**
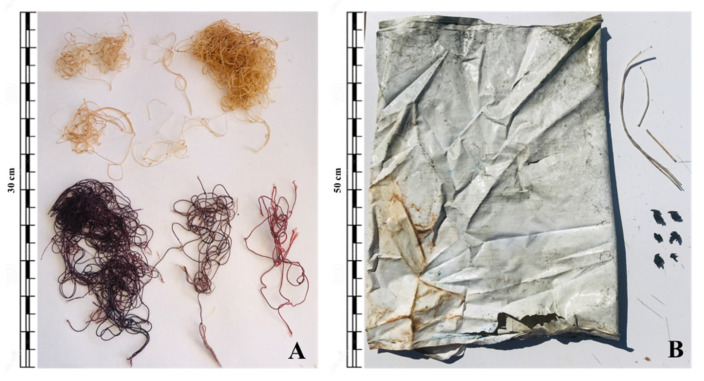
Examples of plastic debris recovered from cetaceans in the Balearic Islands. (**A**) Fishing rope items encountered in the stomach of a necropsied female adult of *T. truncatus* (TL: 3.1 m); (**B**) white plastic bag and plastic packing strap fragments collected from the stomach of a necropsied *P. macrocephalus* (TL: 5.4 m).

In 2021, two female adults of *T. truncatus*, and one male juvenile of *P. macrocephalus*, from the total 30 cetaceans necropsied between 2019 and 2022, presented plastic litter in their digestive tracts, which represents a frequency of occurrence (FO) of 10%. A total of 10 plastic items were obtained, comprising 64.6 g dry mass, with an average dry mass of 12.9 ± 12.7 g per individual, ranging from 0.1 to 58.1 g. In both species, all items were found in the stomach (Figure 2). Curiously, the two specimens of *T. truncatus* presented the same type of litter: one and three pieces of fishing rope, respectively. Instead, plastic litter items identified in the stomach of the *P. macrocephalus* were three plastic packing straps, one from a plastic bag, and two plastic sheets. Of the plastic pieces collected from the gastric contents, the length ranged between 3 cm (fishing rope fragment) and 50 cm (plastic bag). Among the total samples analysed, the most frequently observed plastic shape was thread-like (70%), followed by sheet-like (30%). The dominant colour category of plastic items recovered was white–transparent (*n* = 6), followed by dark (*n* = 3), then light (*n* = 1). All plastic items were included in macroplastics category, and no mesoplastics or microplastics were found. Out of the 27 cetaceans that were found without ingested marine litter, 12 of them presented a totally empty digestive tract; although in 15 specimens, food remains such as fish bones and squid fragments were observed. Moreover, the three specimens that ingested litter showed prey remains (Table 1).

All plastics retrieved were successfully characterised by FT-IR spectroscopy to identify the type of polymer. These analyses detected three different polymers: polypropylene (PP), polyamide (PA), and high-density polypropylene (HDPE). In particular, the dominant polymer types were PP (50%), which is present in plastic sheets and fishery equipment, and PA (40%), which is present in nylons. HDPE (10%), which is mostly used in single-use plastics, was only found in the plastic bag.

### 3.2. Entanglement Analysis

**Figure 3:**
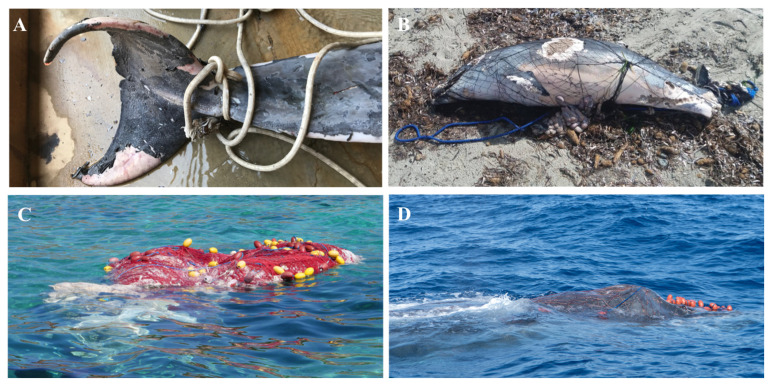
Entanglement cetaceans recorded during the period 2019–2022 around the Balearic Islands. (**A**) *T. truncatus* (TL: 2.3 m) found dead floating while adrift in the south-eastern coast of Mallorca, with a large rope entangled in the caudal peduncle. (**B**) *S. coeruleoalba* (TL: 1.5 m) found dead in the south of Menorca, totally entangled in fishing nets, displaying the impact of by-catching. (**C**) *P. macrocephalus* found in the south-western coast of Mallorca, in an advanced state of decomposition, but totally entangled. (**D**) *M. novaeangliae* (TL: 14 m) found alive in the west coast of Mallorca, with float line entanglements around its dorsal fin, flippers, and caudal peduncle.

In the present study, a total of seven specimens, from four different species, were affected by entanglement between 2019 and 2022 (FO of 7.3%). The other 96 specimens showed no evidence of entanglement, and five specimens were not able to be evaluated for entanglement due to their advanced state of decomposition. Injuries from external entanglement were indicated either by direct observation of gear on the cetacean or scarring that is consistent with wounds from fishing lines (Figure 3). In some cases, due to the advanced state of decomposition, the total length, sex, or age category could not be registered in entangled cetaceans. These seven animals consisted of three *T. truncatus,* two *S. coeruleoalba,* one *P. macrocephalus,* and one *M. novaeangliae*. The vast majority of these species are regularly present in the Mediterranean Sea; however, humpback whales are considered extremely rare in this zone, with fewer than ten confirmed sightings in the last 100 years [44]. Four individuals, one male juvenile of *T. truncatus* (TL: 1.8 m), one *S. coeruleoalba*, one *P. macrocephalus*, and one subadult of *M. novaeangliae* (TL: 14 m) were found dead and stranded with evidence of fishing line entanglements. Of these, two specimens, *T. truncatus* and *P. macrocephalus*, died due to the entanglement. In July 2019, a *S. coeruleoalba* was found alive with its caudal fin entangled in a fishing line; however, the animal was released by the same individuals who had given notice of the animal’s predicament without waiting for the arrival of the rescue centre technicians. Notably, in May 2022, a *M. novaeangliae* was first reported alive with float line entanglements around its dorsal fin, flippers, and caudal peduncle by a citizen, thus prompting the rapid deployment of expert divers onsite in order to free the whale from the nets (Figure 3). One week later, the whale appeared dead on the Valencian coast, with clearly visible scars from the wounds. Moreover, two *T. truncatus* (one male juvenile (TL: 1.7 m) and one adult) and one male juvenile of *S. coeruleoalba* (TL: 1.5 m) were found with their entire bodies entangled in a fishing line. Furthermore, both of these juvenile specimens showed the same lesions, which were the result of clean-cut amputations of their tail flukes due to the impact of by-catching. Finally, the adult of *T. truncatus* (TL: 2.3 m) was found floating while adrift with a large rope entangled in the caudal peduncle, and it was anchored to a big weight at the bottom of the sea (Figure 3). The carcass was opened, and its digestive tract was analysed for plastic ingestion. No remaining foreign material or line was evident in the stomach, nor were food remains, such as fish bones and squid fragments, observed. Out of the items analysed by FT-IR, 60% were PA, 20% were HDPE, and 20% were polyvinylchloride.

## 4. Discussion

Globally, the visible evidence of interactions between marine mammals and marine litter is well documented [9,45,46]; although, on a local level, research is still being carried out on this matter. To address this issue, the present paper provides evidence of the impact of plastic debris, in terms of ingestion and entanglement, in stranded cetaceans, in the Balearic Islands, for the first time. The collected data allow for the improvement of current knowledge concerning the impacts of plastic on cetaceans in the western Mediterranean Sea, in addition to highlighting the importance of working with apex predators, such as cetaceans, for their multiple roles in the conservation of marine environments and ecosystems. Cetaceans can serve as (1) sentinel species to indicate environmental conditions, (2) umbrella species to benefit the conservation of co-occurring species, and (3) flagship species to attract public and political attention [47,48,49,50].

Since 1998, the organisation Servei de Protecció d’Espècies del Govern de les Illes Balears and COFIB, in collaboration with Marineland-Mallorca and Fundación Palma Aquarium, have attended to more than a thousand dead and alive cetaceans along the Balearic Islands’ coastline. Cetaceans from 2019 to 2022 were analysed by monitoring the cetaceans’ gastrointestinal content and entanglement events; the results revealed that 10% of the cetaceans examined showed plastic ingestion, and 7.3% were affected by entanglement.

Cetaceans usually forage at a depth where direct observations of their feeding habits are notoriously complicated [15]; thus, cetacean strandings present a unique opportunity to obtain knowledge concerning the feeding features and gastric content of these animals. Moreover, although this is an indirect method, the digestive content analysis of stranded cetaceans has been well-established for studying marine debris ingestion [9,25]. The current occurrence of plastics in the digestive contents of odontocete [15,24,46,51] and mysticete [52] species has been recorded. Data collated from worldwide stranding networks (sample sizes of more than 10 animals), show high geographic, intra-, and inter-specific variation in terms of the rate of debris ingestion, ranging from 0% to 31% [25]. For example, in the Southwest Atlantic coast, Denuncio et al. (2011) [53] found a FO of 30.1% in 106 bycaught individuals of *Pontoporia blainvillei* examined in Argentina, whereas the FO reported by the estuarine dolphin *Sotalia guianensis*, in Brazil, was 12% [25]. On the north-eastern Atlantic coast, Lusher et al. (2018) [46] examined a total of 528 cetaceans from 11 different species in Ireland, and 45 (FO 8.5%) had marine debris in their digestive tracts. A slightly lower FO was reported in *T. truncatus* in the UK (FO 4.2%), where only one of the 24 individuals analysed presented plastic debris in their stomach. An even lower FO of 1.4% was reported by Duras et al. (2021) [54], who examined 290 animals (*T. truncatus, S. coeruleoalba* and *Ziphius cavirostris*) in Croatia, and found only four individuals who ingested marine debris. Interestingly, in the Mediterranean, the obtained data revealed a higher FO than those values reported by Lacombe et al. (2020) [55] (FO 4.5%). In that study, only four animals, two *T. truncatus*, one *S. coeruleoalba*, and one *D. delphis*, of the 88 stranded cetaceans from the Catalonian coast (western Mediterranean Sea), in the period 2012−2019, were found to contain marine debris (three plastic items and one fishing rope). In contrast, Alexiadou et al. (2019) [15] examined 34 individuals of seven odontocete species stranded along the Greek coasts between 1993 and 2014; they displayed higher levels of debris ingestion (FO 26.5%), with nine individuals affected. The results of this final study were extremely concerning; *P. macrocephalus* had the highest FO for plastic (FO 60%), specifically, one individual in particular, who was stranded in 2006, contained 135 items, predominantly plastic bags.

As demonstrated in the abovementioned studies, debris ingestion is increasingly recognised as an important threat to cetaceans [25]. Various authors have attempted to answer the question concerning why marine organisms ingest debris. Several hypotheses have been formulated to explain this phenomenon, including: confusing marine debris with prey [56,57,58]; prey being in close proximity to debris; not echolocating when finally approaching prey [59,60]; juvenile inexperience [29,61]; the prey capture mechanism in beaked whales [62]; playful and curious behaviour [29]; disease factors; and stranding events [63]. Scientific authors have suggested that the mistaken ingestion of debris, due to a resemblance to the cetacean’s preferred prey, is unlikely to occur in odontocete cetaceans because it is widely accepted that toothed whales use a highly sophisticated echolocation system for foraging [63,64,65]. Given this, any object that is different from fish or cephalopods should not normally be ingested by odontocetes; however, in some cases, this has occurred. In searching for a possible explanation, diverse authors have shown that the disease of the central nervous system (CNS) is a significant risk factor for the ingestion of marine debris that does not pertain to the normal diet of odontocetes [55,63]; nonetheless, in our study, the ingestion of marine debris cannot be directly related to the impairment of nervous function, since nervous samples were not analysed.

Cetaceans can be killed by plastic ingestion because of gastric impaction and perforation or as a result of the associated lesions [66]. In only one case, the cause of death was presumed to be stomach perforation and obstruction caused by a large plastic bag and plastic packing straps. In the *P. macrocephalus* male juvenile, a small opening was made in the abdominal cavity and squid beaks were found on the exterior surfaces of the small intestines, which were loose within the peritoneal cavity. It must be noted that *P. macrocephalus* ingest their prey whole via suction [67,68,69], thus making them prone to the accidental ingestion (passive) of debris found adjacent to their prey. In the case of *T. truncatus* and *S. coeruleoalba*, which also presented plastic ingestion, as inhabitants of shallow coastal water, they share the coastal environment with humans, which results in increased vulnerability due to anthropogenic activities, mainly fishery activities; hence, finding fishing net fragments inside both stomachs was considered evidence of how fisheries impact cetaceans [9,25]. In this sense, residual materials and ghost nets should require more attention for impact monitoring and future conservation policies. Moreover, cetaceans that die from ingesting debris have been observed to swim with difficulty in the days prior to death, which may increase the risk of being struck by ships or boats [15]. Alexiadou et al. (2019) [15] showed that half of ship-struck cetaceans stranded in the Greek Seas (Eastern Mediterranean Sea) had ingested plastic. For this reason, death resulting from plastic ingestion may be more frequent than direct mortalities from confirmed gastric obstructions or perforations would suggest. Although no signs of ship collisions were observed in our study, the importance of exhaustive external examinations in a stranded event must be highlighted.

The Mediterranean Sea is considered to be one of the most polluted areas in the world [70,71,72] with research showing that plastic is the most commonly encountered debris type in these waters, from coastal shelves to offshore deep environments [73,74,75,76]. Lost or discarded fishing gear is considered to be one of the most important sources of debris in the marine environment, it is often found along continental shelves and remote islands [9], and it is mainly composed by PP and PA [77]. Land-based sources of debris mainly include packages, bags, footwear, cigarette lighters, and a wide range of household items that are dumped in the sea; these are mostly made of HDPE. However, sourcing the origin of ingested plastic items is difficult. In the present study, FT-IR spectra of the samples showed that the majority of debris found inside the stomachs of the examined individuals were PP, PA, and HDPE plastics; therefore, in future assessment programs, spectroscopic analysis must be used to determine the nature of the plastic polymers in order to better define the nature and source of contamination in bioindicator species.

Marine debris affects cetaceans by entanglement, except in instances when ingestion has occurred [4,9,25,50]. Entanglement in fishing nets or ghost nets can be difficult to assess in cetaceans as they spend most of their time underwater [78]. Nevertheless, it is known that the lethal effects of entanglement include death by drowning, whereas sub-lethal ones include skin lesions, flipper amputations, compromised feeding, limited predator avoidance capabilities, and reduced reproductive capacity and growth, which eventually lead to decreased fitness [79,80,81]. Ascertaining which cetaceans died due to entanglement as a result of bycatching due to fisheries can be complicated. More than 20 years ago, Kirkwood et al. (1997) [82] published that 40.9% of the cetaceans (*P. phocoena* and *D. delphis*) stranded around the coasts of England and Wales between 1990 and 1995 died by entanglement in fishing gear. During the period 2019–2022, seven specimens (7.3%) of the 108 that were attended to, from four different species (*T. truncatus, S. coeruleoalba, P. macrocephalus*, and *M. novaeangliae*), were affected by entanglement in the Balearic Islands. Three male juvenile specimens (two *T. truncatus* and one *S. coeruleoalba*) skin lesions related to the entanglement, including a mutilated tail. According to Lusher et al. (2018) [46], this type of phenomena is usually linked to interactions between fisheries, where fishers release the dead animals from their fishing gear. Moreover, a carcass of *P. macrocephalus* was found entangled in illegal driftnets in July 2020. Each year, illegal driftnetting causes the entanglements and deaths of *P. macrocephalus* in different Mediterranean regions [83,84]. The *P. macrocephalus* Mediterranean population, which is isolated from the Atlantic population, as shown by genetic evidence [85,86], is considered to be “Endangered”, according to the IUCN Red List. Indeed, in the last half century, the Mediterranean population has faced drastically declined, counting less than 2500 mature individuals [87,88,89]. The main threat faced by this species is entanglement in large-scale fishery driftnets [90]; in 1994, this was classified as “a concerning matter” by the International Whales Commission (IWC). An even more exceptional case was reported in June 2020, when an adult of *T. trucatus* was found dead with a heavy weight attached to its tail, causing it to become fixed to the seabed. The necropsy revealed that the animal died by drowning and had abundant amounts of undigested prey in the stomach. As already suggested by Fertl (1997) [91], cetacean interactions with trawls are complex, in part, because both fishermen and cetaceans are drawn to areas of high prey density. In addition, within such areas, cetaceans are likely to be attracted to trawling activities because they make it easier for the animals to exploit a concentrated food source. Regarding this issue, the relationships between cetaceans and trawls need to be further investigated to determine what effects the fisheries have on the ecology and population status of the cetacean species involved. In this study, only two individuals could be released alive from the nets. The first one, a *S. coeruleoalba*, was stranded in July 2019, presented nonlethal injuries, and never appeared again. Although these negative events may not generally result in acute-lethal interactions, such as in the abovementioned case, damage to the dorsal fin can trigger animal health impairments that disturb vital functions [92]. Such interactions may make catching prey more complicated, leading to starvation or enhanced energy loss while searching for prey. Additionally, the injuries caused by fishing apparatus may lead to infectious diseases or a severe inflammation process that could produce death. The second case involving the release of an animal took place on May 2022, when a *M. novaeangliae* was spotted completely entangled on the eastern cost of Mallorca (Spain) at 4.8 km off Cala Millor. The cetacean was generally quiet, showing a general status of debilitation/weakness, numerous skin lesions, and a low breathing rate. Experienced divers collaborated with one another to ensure that the mesh was removed, and the whale was freed; however, the carcass was found floating on 26 May 2022 in Tavernes de la Valldigna (Valencian Community, Spain), and eventually washed ashore one day later. The carcass displayed scarred lacerative lesions on the skin, and nets (probably pelagic driftnets) around its mouth indicated entanglement and interactions with fisheries. Furthermore, barnacles and whale louse crustaceans that completely covered the animal suggested that this whale was slowly moving for many days before its death. It must be noted that although the entanglement events of *M. novaeangliae* are well known in the Pacific [93] and the Atlantic Ocean [94,95], they were considered extremely rare in the Mediterranean Sea. Indeed, this is the first study to investigate this issue, with a focus on this species, in the Balearic Islands.

## 5. Conclusions

Marine debris poses a critical issue to marine habitats and wildlife, often with fatal consequences. The ingestion of, and entanglement in, plastic litter are recognised as the most significant threat to the survival of cetacean species and populations globally. To the best of our knowledge, this is the first study to demonstrate the presence of plastic ingestion, and incidences of entanglement, with regard to cetaceans from the waters surrounding the Balearic Islands.The specimens studied were from six different species: *S. coeruleoalba*, *T. truncatus*, *G. griseus*, *B. physalus*, *P. macrocephalus*, and *M. novaeangliae*, the latter of which constituted the first case of entanglement, for this species, in the Balearic Islands. The obtained results demonstrate a frequency of occurrence of 10% and 7.3% for plastic ingestion and entanglement, respectively. In this study, the death of only one individual (*P. macrocephalus*) could be directly related to plastic ingestion. The characterisation of the plastics ingested, and the plastics that the animals became entangled in, was carried out in accordance with the following categories: size, shape, colour, and polymer type. Characterising the plastics in this manner could help with the recognition of the plastics’ principal origin. Nevertheless, targeted efforts for the standardisation of protocols for collecting data are required. This study provides a baseline that will serve as a reference point for a future monitoring framework.

## Figures and Tables

**Table 1:** Information about the stranded cetaceans whose digestive tract contents were analysed for plastic debris.

Code	Stranding Date	Total Length (m)	Age Class	Sex ^1^	Presence of Prey Remains ^1^	Debris Found	Debris Type
*S. coeruleoalba 1*	09.06.19	2.0	Adult	f	n	n	--
*S. coeruleoalba 2*	17.10.19	1.6	Juvenile	m	y	n	--
*S. coeruleoalba 3*	24.10.19	1.9	Adult	--	y	n	--
*S. coeruleoalba 4*	29.11.19	2.3	Adult	f	y	n	--
*S. coeruleoalba 5*	20.12.19	1.0	Calf	m	n	n	--
*S. coeruleoalba 6*	06.02.20	1.9	Adult	--	y	n	--
*S. coeruleoalba 7*	13.05.20	1.4	Juvenile	m	n	n	--
*S. coeruleoalba 8*	12.12.20	1.7	Juvenile	f	n	n	--
*S. coeruleoalba 9*	02.03.21	2.3	Adult	f	y	n	--
*S. coeruleoalba 10*	02.03.21	2.0	Adult	f	y	n	--
*S. coeruleoalba 11*	11.03.21	2.0	Adult	f	n	n	--
*S. coeruleoalba 12*	20.07.21	2.0	Adult	m	n	n	--
*S. coeruleoalba 13*	23.07.21	1.9	Adult	f	n	n	--
*S. coeruleoalba 14*	26.07.21	2.0	Adult	f	y	n	--
*S. coeruleoalba 15*	26.02.22	1.9	Adult	f	y	n	--
*T. truncatus 1*	11.08.19	1.7	Juvenile	m	n	n	--
*T. truncatus 2*	16.06.20	2.3	Adult	m	y	n	--
*T. truncatus 3*	30.12.20	2.3	Adult	m	y	n	--
*T. truncatus 4*	14.01.21	3.2	Adult	f	y	y	Fishing ropes
*T. truncatus 5*	05.05.21	3.1	Adult	f	y	y	Fishing ropes
*T. truncatus 6*	01.03.22	2.6	Adult	m	n	n	--
*T. truncatus 7*	20.04.22	1.7	Juvenile	m	y	n	--
*T. truncatus 8*	17.07.22	3.4	Adult	--	y	n	--
*T. truncatus 9*	20.08.22	0.9	Calf	f	n	n	--
*G. griseus 1*	29.03.21	2.6	Juvenile	f	y	n	--
*G. griseus 2*	04.04.22	3.1	Adult	f	y	n	--
*B. physalus 1*	26.01.19	15.5	Adult	f	n	n	--
*P. macrocephalus 1*	16.05.21	5.4	Juvenile	m	y	y	Plastic bag, Packing straps, Plastic sheets
Unknown	04.05.22	1.3	Juvenile	m	y	n	--
Unknown	01.09.22	83.0	Calf	f	n	n	--

^1^ Abbreviations: f (female), m (male), y (yes), n (no).

## Data Availability

Researchers wishing to access the data used in this study can make a request to the corresponding author: Antoni.sureda@uib.es.
